# The Chromatin Regulator *Ankrd11* Controls Palate and Cranial Bone Development

**DOI:** 10.3389/fcell.2021.645386

**Published:** 2021-04-29

**Authors:** Daniela Marta Roth, Pranidhi Baddam, Haiming Lin, Marta Vidal-García, Jose David Aponte, Sarah-Thea De Souza, Devyn Godziuk, Adrianne Eve Scovil Watson, Tim Footz, Nathan F. Schachter, Sean E. Egan, Benedikt Hallgrímsson, Daniel Graf, Anastassia Voronova

**Affiliations:** ^1^School of Dentistry, Faculty of Medicine and Dentistry, University of Alberta, Edmonton, AB, Canada; ^2^Department of Cell Biology & Anatomy, Alberta Children’s Hospital Research Institute, University of Calgary, Calgary, AB, Canada; ^3^Department of Medical Genetics, Faculty of Medicine and Dentistry, University of Alberta, Edmonton, AB, Canada; ^4^Cell Biology Program, Hospital for Sick Children, Toronto, ON, Canada; ^5^Department of Molecular Genetics, University of Toronto, Toronto, ON, Canada; ^6^Department of Cell Biology, Faculty of Medicine and Dentistry, University of Alberta, Edmonton, AB, Canada

**Keywords:** KBG syndrome, epigenetic regulation, craniofacial development and malformations, intramembranous ossification, bone remodeling, cleft palate, neurodevelopmental disorders, chromatin regulation

## Abstract

Epigenetic and chromatin regulation of craniofacial development remains poorly understood. Ankyrin Repeat Domain 11 (*ANKRD11*) is a chromatin regulator that has previously been shown to control neural stem cell fates via modulation of histone acetylation. *ANKRD11* gene variants, or microdeletions of the 16q24.3 chromosomal region encompassing the *ANKRD11* gene, cause KBG syndrome, a rare autosomal dominant congenital disorder with variable neurodevelopmental and craniofacial involvement. Craniofacial abnormalities include a distinct facial gestalt, delayed bone age, tooth abnormalities, delayed fontanelle closure, and frequently cleft or submucosal palate. Despite this, the dramatic phenotype and precise role of *ANKRD11* in embryonic craniofacial development remain unexplored. Quantitative analysis of 3D images of KBG syndromic subjects shows an overall reduction in the size of the middle and lower face. Here, we report that mice with heterozygous deletion of *Ankrd11* in neural crest cells (Ankrd11^nchet^) display a mild midfacial hypoplasia including reduced midfacial width and a persistent open fontanelle, both of which mirror KBG syndrome patient facial phenotypes. Mice with a homozygous *Ankrd11* deletion in neural crest cells (Ankrd11^ncko^) die at birth. They show increased severity of several clinical manifestations described for KBG syndrome, such as cleft palate, retrognathia, midfacial hypoplasia, and reduced calvarial growth. At E14.5, *Ankrd11* expression in the craniofacial complex is closely associated with developing bony structures, while expression at birth is markedly decreased. Conditional deletion of *Ankrd11* leads to a reduction in ossification of midfacial bones, with several ossification centers failing to expand and/or fuse. Intramembranous bones show features of delayed maturation, with bone remodeling severely curtailed at birth. Palatal shelves remain hypoplastic at all developmental stages, with a local reduction in proliferation at E13.5. Our study identifies *Ankrd11* as a critical regulator of intramembranous ossification and palate development and suggests that Ankrd11^nchet^ and Ankrd11^ncko^ mice may serve as pre-clinical models for KBG syndrome in humans.

## Introduction

Molecular studies in developmental biology have been instrumental in defining gene regulatory and signaling networks that control cell and tissue differentiation to a considerable extent. These studies have revealed that throughout tissue development, specific transcription factors or signaling molecules are often used in a reiterated fashion to elicit discrete, developmental stage-specific cellular responses. Epigenetic mechanisms, defined here as external modifications of chromatin, facilitate these stage-specific responses. Through control of global regulation of gene expression at the various developmental stages (Bannister and Kouzarides, 2011; Budhavarapu et al., 2013), they control cell identity and facilitate stage-specific molecular responses (Barrero et al., 2010; Mirabella et al., 2016). A fitting example for complex tissue development is craniofacial development, which involves the coordinated but often asynchronous development, growth, and maturation of initially independent bone and cartilage structures. Epigenetic control mechanisms help facilitate different bone-related processes: bone development, bone mass accumulation and growth, maintenance and remodeling, and bone loss at different stages (Strobl-Mazzulla et al., 2012; Hu et al., 2014; Marini et al., 2016). The complexity of chromatin regulation controlling DNA accessibility to transcriptional machinery during craniofacial morphogenesis is still poorly understood, although dysregulation of epigenetic mechanisms has been implicated in a number of musculoskeletal and rheumatic diseases (Jeffries and Sawalha, 2015).

The entry point for this study was the association of the chromatin regulator *ANKRD11* (Ankyrin Repeat Domain 11; previously named ANCO-1) with KBG syndrome (OMIM #148050), a rare, autosomal dominant, congenital disorder characterized by a distinct craniofacial gestalt (Morel Swols and Tekin, 2018). *ANKRD11* contains an Ankyrin domain consisting of five Ankyrin repeats, two repression domains, and one activation domain (Zhang et al., 2007), and regulates global gene expression (Gallagher et al., 2015) by interacting with a variety of histone acetylation modifying proteins, such as HDAC3 (histone deacetylase 3) (Zhang et al., 2004; Gallagher et al., 2015) and components of the P/CAF (p300/CBP-associated factor) acetyltransferase complex (Li et al., 2008). While the full-length *ANKRD11* protein acts as a transcriptional repressor, the activation domain elicits transcriptional activation (Zhang et al., 2004, 2007; Li et al., 2008). Moreover, *ANKRD11* interacts with and increases acetylation of p53, potentiating the ability of p53 to act as a transcription factor (Neilsen et al., 2008), which itself is involved in many aspects of craniofacial development (Bowen and Attardi, 2019).

KBG syndrome is associated with heterozygous mutations in *ANKRD11* or micro-deletions of 16q24.3 encompassing *ANKRD11.* While patients with KBG syndrome display a range of phenotypes, a consistent feature is the distinct craniofacial gestalt (Morel Swols and Tekin, 2018). KBG syndrome diagnosis is typically suspected in an individual that displays macrodontia and/or characteristic facial appearance along with two additional criteria, such as palatal abnormalities, hearing loss, short stature, delayed bone age, scoliosis, learning difficulty, etc. (Morel Swols and Tekin, 2018). Delayed closure of a large anterior fontanelle is also frequently observed (Low et al., 2016).

The facial skeleton is mainly formed by neural crest cell-derived intramembranous bones, which develop from mesenchymal condensations at defined ossification centers (Berendsen and Olsen, 2015). During this process, osteochondroprogenitor cells sequentially differentiate into osteoblasts to form intramembranous bone. Osteoblasts deposit initially unmineralized osteoid that subsequently mineralizes (Berendsen and Olsen, 2015). As bone matures, osteoblasts become trapped within and terminally differentiate into osteocytes (St John et al., 2014). Maturation of osteocytes involves loss of organelles and changes in molecular properties (Irie et al., 2000). Newly formed bone matures through a process termed bone remodeling. This term describes the continuous process of bone resorption and bone formation that allows bone to attain its mature shape and optimal mechanical strength (Hadjidakis and Androulakis, 2006). These events require coordinated function of bone-resorbing osteoclasts, bone-depositing osteoblasts, and mature osteocytes. During this process, trabecular bone matures, and many osteocytes are resorbed.

Mineralization of the craniofacial complex begins in the mouse embryo around embryonic day 14 (Flaherty and Richtsmeier, 2018). While the involvement of epigenetics in the components of the ossification process is well documented (Schroeder et al., 2004; Li et al., 2009; Wein et al., 2015), there is little known about the overall contribution of chromatin regulators to craniofacial development.

Palate development describes the process by which the initially vertically growing maxillary appendages, termed palatal shelves, reorient to move above the tongue, grow horizontally and fuse to separate oral and nasal cavities (Kouskoura et al., 2011). The proliferation and differentiation of the palatal mesenchyme are regulated by intricate epithelial-mesenchymal interactions along the anterior-posterior axis. Palatal abnormalities, which occur when any of these required developmental events are disturbed, are relatively frequent and can present as complete, partial, or submucosal clefts. There is good evidence from the mouse for the involvement of epigenetic regulation during palate development (Kuriyama et al., 2008; Seelan et al., 2013; Juriloff et al., 2014) although direct evidence from humans is largely missing (Sharp et al., 2018).

Craniofacial and palate anomalies associated with KBG syndrome patients suggest a direct role for *ANKRD11* during craniofacial development. A homozygous *Ankrd11* missense mutation in mice is lethal in early embryonic stages (E9), preventing studies on craniofacial development with complete loss of *Ankrd11* (Barbaric et al., 2008). As neural crest cells contribute significantly to development of the anterior craniofacial complex, we used a neural crest-specific Wnt1Cre2 Cre-*lox* mouse line (Lewis et al., 2013) to delete *Ankrd11* in the developing neural crest.

We found that homozygous deletion of *Ankrd11* in neural crest (conditional knockout; Ankrd11^ncko^) is perinatal lethal, while mice lacking one copy of *Ankrd11* (conditional heterozygote; Ankrd11^nchet^) survive into adulthood. Adult heterozygous mice recapitulate some of the overall craniofacial phenotypes seen in KBG patients, whereas knockout embryos and pups display increased severity of numerous craniofacial anomalies commonly reported for patients with KBG syndrome (delayed ossification, cleft palate, midfacial hypoplasia, persistent anterior fontanelle, retrognathia). Our study identifies *Ankrd11* as a critical regulator of intramembranous ossification and palate development and suggests that Ankrd11^nchet^ and Ankrd11^ncko^ mice may serve as novel pre-clinical models for KBG syndrome.

## Materials and Methods

### Mice

Animal experiments were approved by the Research Ethics Office at the University of Alberta (Animal Care and Use Committee, AUP1149, AUP2527) in compliance with guidelines set by the Canadian Council of Animal Care. Mice on a C57Bl/6 background were housed in the animal facility at the University of Alberta. Ankrd11^TM 1a(EUCOMM)Wtsi//^IcsOrl mice, in which exon 7 was floxed, were rederived from sperm (EM:07651, the European Mouse Mutant Archive – Infrafrontier) and crossed with Flp recombinase expressing (Jackson Labs) mice to generate Ankrd11^TM 1*c*^ conditional ready mice (Ankrd11^*fl/fl*^) as described (Skarnes et al., 2011). Exon 7 is located within the Ankyrin repeat domain. Ankrd11^*fl*/fl^ mice were crossed with B6.Cg-E2f1Tg(Wnt1-cre)^2*Sor/J*^ (Jackson Labs) (Lewis et al., 2013) mice for neural crest-specific deletion of *Ankrd11* (Ankrd11^ncko^). Deletion of exon 7 results in out-of-frame splicing of exon 6 to exon 8, leading to a pre-mature stop and truncation of *Ankrd11*. Genotyping of mice was performed using Taq DNA Polymerase 2x Master Mix RED (Ampliqon, Denmark) from ear-notch or tissue biopsies. The following primers and PCR conditions were used: (1) for *Ankrd11*: forward, 5′-CTGTCTCAGAGAGGAGAGTGAGGAGGAC-3′; reverse, 5′-TACCTTACACCCTGAGACGGCGTC-3′; 34 cycles of: 94°C-30 s, 62°C-45s, 72°C-60s; (2) for the Cre transgene: forward, 5′-TTCCCGCAGAACCTGAAGATG-3′; reverse, 5′-CCCCAGAAATGCCAGATTACG-3′; Twsg1 internal control forward, 5′-AACAACAATGGCACAACCTAAT-3′, Twsg1 reverse, 5′-ACTTTCTCCCCACCCGTCTA-3′; 35 cycles of: 94°C-15s, 60°C-30s, 72°C-90s.

### Micro-Computed Tomography

Mice were imaged using a MILabs μCT scanner at the School of Dentistry at the University of Alberta. Heads were fixed in 4% paraformaldehyde (PFA) for 24 h, then scanned at the following parameters: voltage = 50 kV, current = 0.24 mA, exposure time = 75 ms, 1600 exposures/full rotation. Scans were reconstructed at a voxel size of 25–35 μm, and the volumes were exported as NifTI-1 files. NifTI-1 files were directly analyzed using Amira software (version 2019.2, Life Technologies). NifTI-1 files were batch-converted to MINC-2 format using a custom made bash script and the MINC toolkit (Vincent et al., 2016).

### Morphometric Analysis

#### Human Morphometrics

We acquired four three-dimensional (3D) scans of confirmed KBG syndrome patients from FaceBase (Samuels et al., 2020). All human subjects provided informed consent for the use of facial image data as approved by Institutional Review Boards at the University of Calgary, University of Colorado, Denver and the University of California, San Francisco. Each scan was non-linearly registered to a dense atlas (27,000 vertices) using the non-rigid iterative closest point method described in Bannister et al. (2020). We used a linear model with the first 400 principal components of facial shape to adjust the dense registered meshes for the effects of age and sex as described in Hallgrímsson et al. (2020). We then compared the mean age and sex-adjusted KBG mesh to the mean non-syndromic mesh. We visualized the differences between means as a heatmap using the meshDist() function in the Morpho package for R (Schlager, 2017).

#### Mouse Morphometrics

We used the MINC toolkit (Vincent et al., 2016) to gather 3D coordinates of 68 anatomical landmarks, from x-ray micro-CT volumes of the cranium of *Ankrd11* and control mice (Supplementary Figure 1). We also placed landmarks on a mouse skull atlas (average) and extracted a surface mesh of its segmented cranium, using marching cubes in VTK (Schroeder et al., 1998) and the MINC toolkit (Vincent et al., 2016).

We imported the specimen landmark files and the atlas landmark data into R (R Core Team, 2020) using the functions tag2array() and tag2lm(), respectively (Vidal-García, 2021). We performed a Generalized Procrustes Analysis (GPA) using the function gpagen() in geomorph (Adams et al., 2020), in order to obtain shape coordinates and remove effects due to size, rotations, and translations in the 3D landmark data. We then calculated group means on the shape coordinates for *Ankrd11* and control specimens, using the function mshape() in geomorph (Adams et al., 2020).

We mapped these 3D shape coordinate means to the atlas mesh and landmark coordinates using the function tps3d() in Morpho (Schlager, 2017). This function uses a thin plate spline method (Bookstein, 1989) to interpolate a target set of landmarks to the reference landmark set (atlas), which then warps the atlas mesh to fit the target landmark data. We visualized morphological differences between the *Ankrd11* and control mean meshes with heatmaps, using the function meshDist() in Morpho (Schlager, 2017).

Three-dimensional segmentation was performed using 3D slicer software. Automated segmentation function was used utilizing island and scissor functions to isolate individual bones. Bone volume was determined using statistics functions.

### Tissue Preparation and Histology

Embryos were dissected from uterus at embryonic days (E) 13.5, 14.5, and 15.5 and collected at birth at postnatal day (P) 0. Embryos were decapitated and heads were fixed overnight at 4°C in 4% PFA. P0 heads were decalcified in 0.5M EDTA overnight before processing. Heads were processed and sectioned as previously described (Baddam et al., 2020). Before further analysis, slides were warmed in a 60°C oven, deparaffinized with xylene, and rehydrated through graded ethanol washes.

#### Histological Analysis

Rehydrated slides were stained with hematoxylin & eosin (H&E) (Harris modified hematoxylin Fisher SH30, eosin Sigma E4382), Alcian blue (1% Alcian blue solution, pH 1, 0.1N hydrochloric acid rinse, nuclear fast red counterstain), picrosirius red (Direct red 80 Fisher B21693, picric acid ACPchem P-2095), or Tartrate-Resistant Acid Phosphatase (TRAP). Briefly, TRAP staining was performed by incubating slides in pre-warmed enzymatic TRAP Staining Solution containing Fast Red Violet LB salt (Sigma F-331) at 37°C for 40 min, or until the control slide is slightly overstained. Slides were then counterstained with 0.02% Fast Green (Sigma F-7252). After staining, slides were dehydrated, cleared with xylene, and mounted with Permount (Fisher SP-15).

#### Immunofluorescent Analysis

Immunofluorescent staining was performed as described previously (Malik et al., 2020). Details of primary and secondary antibodies and dilutions used are summarized in Supplementary Table 1. Images were either taken on an Olympus IX73 microscope using 10x, 20x, or 40x objectives to photograph in a single plane and images were captured using the included cellSens Dimension program. Analysis was performed on at least three independent biological replicates; a representative image is shown. Polarized light images were acquired using a polarizer and analyzer cube (Olympus).

### Image and Statistical Analysis

Density of cells forming the palatal shelves was quantified at E13.5 using DAPI nuclear stain (not shown). Semi-automated cell counting was performed in ImageJ on spatially defined regions within the palatal shelves (oral or nasal domains).

The number of immunostained Ki67-positive cells were counted on a minimum of six separate images per age and genotype from a palatal shelf. Sections were from a minimum of five different embryos from at least three different litters. The region counted is indicated in Figure 5 with white dotted lines. Counting was automated in ImageJ using thresholding, watershed and particle analysis functions. Values presented in the graph are number of Ki67 positive cells divided over DAPI positive cells to normalize for hypoplasticity of shelves. Quantification of Runx2 and Sp7 domains at E12.5/13.5 was performed similarly, with values reported as percent of a defined field of view occupied by the stain and/or number of positive cells within the field of view. At least 3 (E14.5) or 6 (P0) sections were analyzed from 3 to 5 mice per genotype from at least three different litters. Statistical significance was determined via an unpaired two-tailed *t-*test.

### *In vitro* Culture

Primary osteoblast cultures were established from calvaria for the gene expression time-course experiment. Calvaria were dissected and cut into small fragments, discarding cranial suture tissue. Calvarial bones were dissected into HBSS (Sigma H-9394) and used for osteoblast isolation using a modified protocol (Bakker and Klein-Nulend, 2012; Perpétuo et al., 2019). HBSS was replaced with 0.25% trypsin for 10-min digestion at 37°C, washed in αMEM (Gibco 12561-056), and digested twice with 0.2% Collagenase Type II solution (Worthington 4176) for 30 min each. Final digestion product was collected. Remaining bone pieces were washed with αMEM – solution was added to final digest and bone pieces were rinsed three times with cCM (complete culture medium: αMEM supplemented with Penicillin-Streptomycin [Gibco 15240-062], ascorbate [100 μg/mL, Sigma, A-8960], 10% Fetal Bovine Serum [Sigma F-1051]), then transferred to 75 cm^2^ flasks containing cCM. Final digest was centrifuged for 5 min at 1500 *g* and the pellet was resuspended in 1 mL αMEM per calvarium. Cell suspension was combined with bone pieces in flask and incubated at 37°C and 5% CO_2_ in air until confluent. cCM media changed every 3 days.

Once confluent, cells were trypsinized and seeded in a 12-well plate at 5–10 × 10^4^ cells per well. Osteogenic medium (αMEM supplemented with ascorbate [50 μg/mL] and 2 mM β-glycerophosphate [Sigma G-5422]) was added when cells reached 80–90% confluency. Medium was changed every 3 days. Cells from three wells were harvested using Trizol (Invitrogen 15596026) at days 0, 3, 6, 9, 12, 15 for mRNA isolation and RT-qPCR quantification. Three independent experiments with two mice each were performed with three wells/time-point/experiment. For mRNA isolation, wells from within an experiment were combined. A representative result is shown.

### RT-qPCR

RNA extraction, cDNA synthesis, and quantitative RT-qPCR were performed as previously described (Malik et al., 2020) using appropriate primer pairs normalized to *36B4* as a reference gene (see Supplementary Table 2). Fold change was calculated using 2^–ΔΔ^ ^Ct  method^ (Livak and Schmittgen, 2001). MIQE guidelines were followed (Bustin et al., 2009).

## Results

### KBG Syndrome Patients and Heterozygous Neural Crest-Specific *Ankrd11*-Mutant Mice (Ankrd11^nchet^) Share Craniofacial Features

**FIGURE 1:**
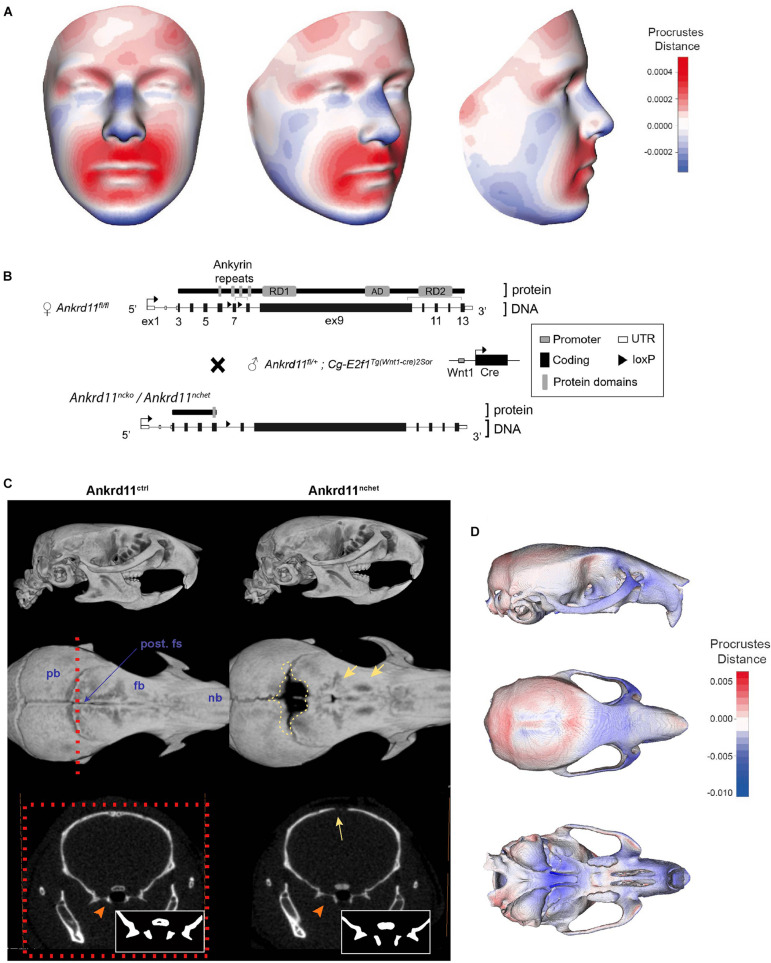
KBG syndrome patients and Ankrd11^nchet^ mice share craniofacial similarities. **(A)** Mesh morphometric analysis of 4 KBG syndrome patient 3D images. Images shown are a mean representation of craniofacial changes on a reference face. Heat map representation in Procrustes distance (relative to superimposition). Red color indicates positive change in size relative to reference mesh and blue indicates negative size difference. White color indicates no change in highlighted region. **(B)** Brief summary of gene targeting and breeding strategy to produce Ankrd11^nchet^ mice, shown in panels **(C,D)**, and Ankrd11^ncko^ mice. **(C)** Reconstructions of μCT scans of Ankrd11^ctrl^ (Ankrd11^*wt/fl*^) and Ankrd11^nchet^ mice. Top panel: lateral view of whole skull. Middle panel: Superior view of frontal bone; mandible and cranial base are clipped out of plane. Red dotted line indicates plane of sectioning for lower row of panels. Yellow dotted line highlights calvarial defect surrounding posterior frontal suture in Ankrd11^nchet^ mice. Yellow arrows point to regions of hard tissue anomalies in Ankrd11^nchet^ frontal bone. Lower panels: orthogonal slice through posterior frontal suture indicating pterygoid abnormalities (orange arrow) and open posterior frontal suture (yellow arrow). Inset in lower right corner of images is high contrast representation of cranial base structures affected in image. pb, parietal bone; fb, frontal bone; nb, nasal bone; post. fs, posterior frontal suture. **(D)** Mesh morphometric analysis of three Ankrd11^nchet^ mouse skulls. Images shown are a mean mesh of three Ankrd11^nchet^ skulls relative to a mean mesh of five Ankrd11^ctrl^ skulls. Note: mandibles were excluded from analysis due to the variable position with respect to the skull. Colors depict Procrustes distances between Ankrd11^ctrl^ and Ankrd11^nchet^ mean meshes, with blue indicating negative values (Ankrd11^nchet^ values fall within Ankrd11^ctrl^ mesh) and red indicating positive values (Ankrd11^nchet^ values fall outside of Ankrd11^ctrl^ mesh). White values indicate no changes, meaning the vertices for these anatomical regions in both mice are very close.

Three-dimensional facial images from four genetically confirmed KBG syndrome patients were combined and mapped against a reference face (Figure 1A) (Hallgrímsson et al., 2020). The resulting mesh heatmaps revealed a consensus hypoplastic mid-and lower faces, with an expansion of the upper third of the craniofacial complex. The *Ankrd11* neural crest-specific haploinsufficient deletion was generated by crossing *Ankrd11* floxed mice with Wnt1Cre2^+^ mice (Ankrd11^nchet^) (Figure 1B). Analysis of μCT scans from mice Ankrd11^nchet^ mice revealed distinct changes to several craniofacial structures (Figure 1C): a persistent open fontanelle, a unique ossification defect in the posterior frontal suture, a slight change in pterygoid bone morphology evident from coronal ortho-slices anterior to the coronal suture (Figure 1C, lower panels). The angle of medial aspect of the pterygoid bone relative to ventrolateral was altered in Ankrd11^nchet^ mice. Comparative mesh heatmaps of Ankrd11^nchet^ and control scans revealed growth alterations in comparable regions as described for the human faces: reduced facial width, hypoplasia of midface, nasal region, and an expanded cranial vault (Figure 1D). Note: mandibles were omitted from analysis because of their variable position with respect to the skull.

### Neural Crest Specific Deletion of *Ankrd11* (Ankrd11^ncko^) Results in Severe Craniofacial Phenotypes

While pups lacking both copies of *Ankrd11* in the neural crest (Ankrd11^ncko^) died at birth, precluding our analysis at the postnatal and adult stage, visual inspection of the neonatal head at postnatal day 0 (P0) revealed partially open eyelids, variable midfacial hypoplasia, reduction of calvarial growth (evident as reduction in calvarial microvasculature), loss of pigment on the nose, fully penetrant cleft palate, and a smaller tongue relative to control mice (Supplementary Figure 2).

### Ankrd11^ncko^ Mice Exhibit Severe Bone Growth Defects

**FIGURE 2:**
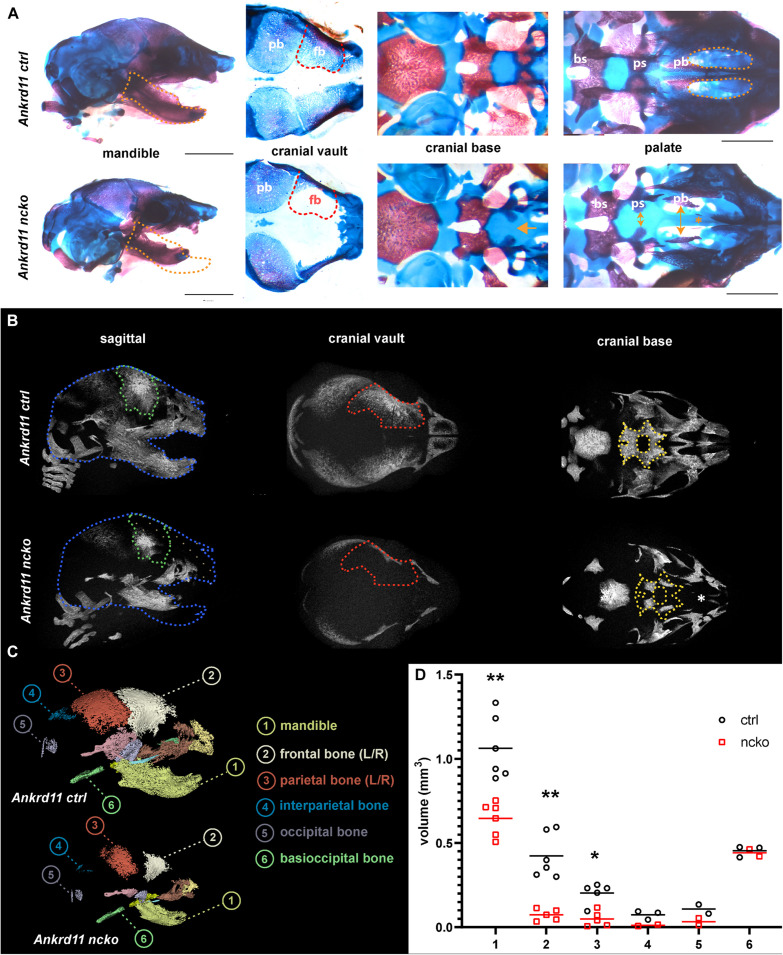
Ankrd11^ncko^ mice have compromised ossification in craniofacial structures at birth. **(A)** Wholemount images of postnatal day 0 (P0) skull skeletal preparations, stained with Alizarin red and Alcian blue. Left panels: Orange dotted lines outline Ankrd11^ctrl^ (Ankrd11^*fl/fl*^) mandible to indicate micrognathia in the mutant. Middle left panels: Red dotted lines outline Ankrd11^ctrl^ (Ankrd11^*fl/fl*^) frontal bone to indicate its smaller size in the mutant. Middle right panels: Yellow arrows indicate extra space between presphenoid and palatine bones of the cranial base. Yellow asterisk to the right highlights clefting of hard palate in Ankrd11^ncko^ mouse. Right panels: magnified view of palate. bs, basisphenoid bone; ps, presphenoid bone; pb, palatine bone. Scale bar represents 2 mm, third column represents 1 mm. **(B)** μCT scans of P0 Ankrd11^ncko^ and Ankrd11^ctrl^ skulls reconstructed in Amira. Blue dotted lines: Ankrd11^ctrl^ skull outline. Green dotted lines: control frontal bone outline. Tan dotted fill in Ankrd11^ncko^: negative space between control mouse and mutant. Red dotted lines: control frontal bone outline. Yellow dotted lines: control basisphenoid and presphenoid hard tissue outline. White asterisk indicates reduction in palatine bone ossification in the mutant mouse. **(C)** representative segmentation of individual bones from μCT scans of P0 Ankrd11^ncko^ and Ankrd11^ctrl^ skulls. **(D)** Quantification of segmented bone volumes (mm^3^) from Ankrd11^ncko^ and Ankrd11^ctrl^ pups (independent two-sample *t*-test assuming unequal variances, **p < 0.001, *p < 0.05, *n* = 6). Segmentation and volume analysis performed using 3D Slicer.

Skeletal preparations of P0 neonates demonstrated an underdeveloped midface and severe micrognathia in Ankrd11^ncko^ mice when compared to littermate controls (Figure 2A). A cleft palate was evident, as was the reduced ossification of the palatine bones and the anterior cranial base (presphenoid and sphenoid) (Figure 2A). Micro-computed tomography (μCT) showed that all intramembranously formed orofacial bones were reduced in size, resulting in underdeveloped midface and mandible (Figure 2B). Ossification of the anterior cranial bones was similarly severely stunted, and bones failed to cover the majority of the cranium at birth when compared to control mice (Figure 2B). Ossification of the anterior cranial base (presphenoid and basisphenoid) was similarly reduced. Several of the primary ossification centers failed to expand and formation of the pterygoid wings was largely missing. In contrast, more posterior, mesoderm-derived components of the cranial base (basioccipital bone) appeared unaffected. Quantification of the volumes of isolated craniofacial bones confirmed 39% reduction of the mandible (*p* < 0.001), 83% reduction of the frontal bone (*p* < 0.001), and 76% reduction of parietal bone (*p* < 0.05), with interparietal, occipital and basioccipital bones being not significantly different (Figure 2C).

### Ankrd11^ncko^ Mice Display Hypoplastic Palatal Shelves and Cleft Palate

**FIGURE 3:**
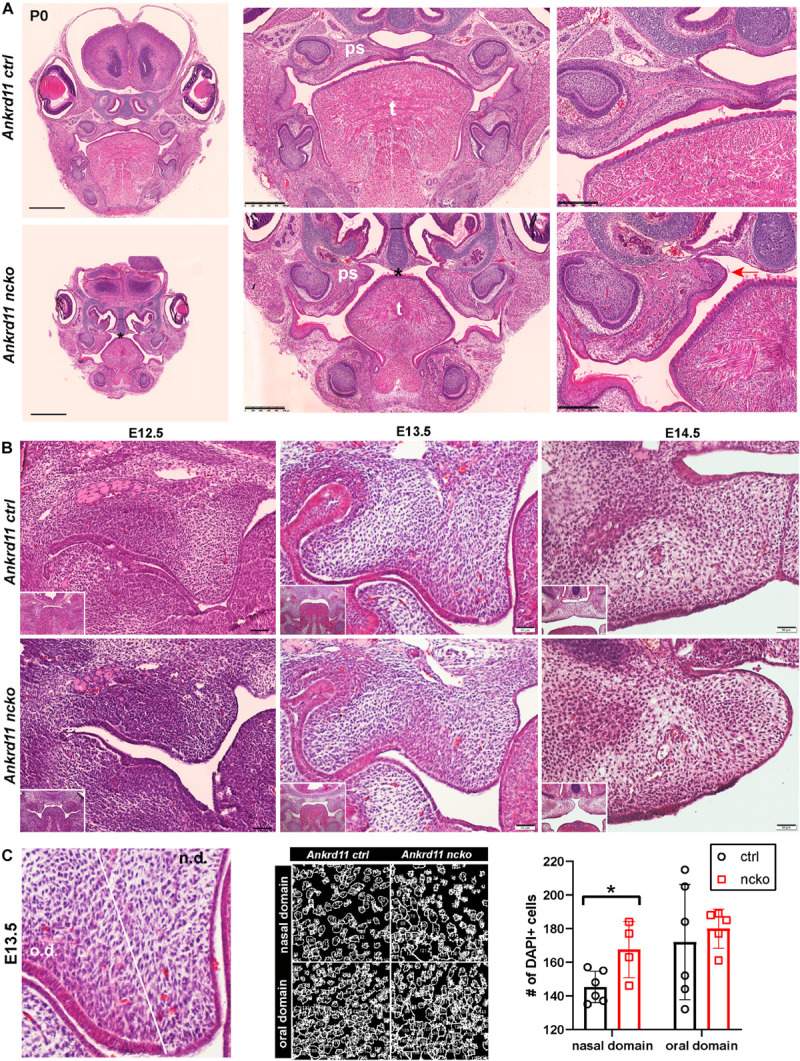
Ankrd11^ncko^ mice display craniofacial changes early in development. Hematoxylin and eosin (H&E) stain. **(A)** Representative images of P0 coronal sections of Ankrd11^ctrl^ (top) and Ankrd11^ncko^ (bottom) skulls. Note relative size difference between heads. Black asterisk indicates absence of complete palatine boundary separating oral and nasal cavities in Ankrd11^ncko^ image. Second column images demonstrate overall differences in oral cavity, including smaller tongue and cleft palate. Differences in plane of section in mandible are due to retrognathia in Ankrd11^ncko^ mice. Right panels focus on palatal shelves, with red arrow indicating unfused advancing medial edge of palatal shelf in mutant mouse. t, tongue; ps, palatal shelf; *cleft palate. Scale bar indicates: 1 mm (first column), 500 μm (second column), and 250 μm (third column). **(B)** Coronal sections of palatal shelves at E12.5, E13.5, and E14.5. Inset in lower right corner of each view is lower magnification image of same mouse, showing overall organization of oral cavity in each mouse. *n* = 3 from 3 litters. Scale bar represents 50 μm. **(C)** Analysis of cell density based on DAPI stain in E13.5 palatal shelves separated into oral and nasal domains. Left: representative H&E stain. Middle: ImageJ-generated density profile showing individual cells. Right: Statistical representation of analysis showing increased cell density in the nasal domain of Ankrd11^ncko^ palatal shelves (independent two-sample *T*-test assuming unequal variances, *p < 0.05). n.d., nasal domain; o.d., oral domain.

Histological analysis confirmed the notable size difference of the head and the cleft palate phenotype (Figure 3A). All major structures were present and developed to a recognizable stage, with exception of the tongue and palatal shelves that remained hypoplastic (Figure 3A). Note that due to retrognathia, mandibular structures on the sections appear more anterior. The developing palatal shelves were hypoplastic and dysmorphic already at embryonic day 12.5 (E12.5) (Figure 3B). Hypoplasia of the tip of the palatal shelf remained evident at later developmental stages. Elevation of palatal shelves appeared normal, but hypoplastic shelves did not meet to fuse, resulting in failure to separate oral and nasal cavities (Figure 3B). Analysis of cell density in the oral and nasal domains of the palatal shelves at E13.5 revealed a 15% increase in cell density in the nasal domain only (*p* < 0.05), with no differences observed in the oral domain (Figure 3C).

### Ankrd11 Expression Is Associated With Early Bone Development and Bone-Lining Cells

**FIGURE 4:**
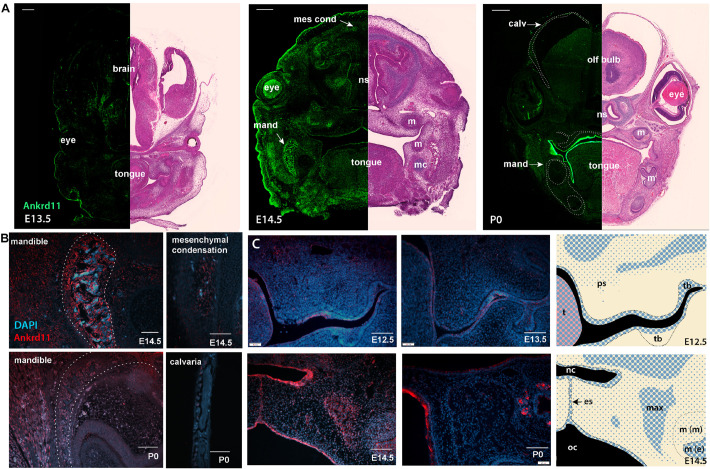
Ankrd11 is expressed in embryonic craniofacial tissues. Expression pattern of Ankrd11 on immunofluorescent staining of Ankrd11^ctrl^ coronal paraffin sections. **(A)** Split view of coronal sections of C57/BL6 mice immunostained for Ankrd11 (left, green) or processed for H&E for morphological orientation (purple, right) at ages E13.5, E14.5 and P0. ns, nasal septum; mand, mandible; m, molar; mc, Meckel’s cartilage; olf bulb, olfactory bulb of brain; calv, calvaria. White dotted lines on P0 immunofluorescent panel outline bony structures of the craniofacial complex including calvaria, maxillary/palatine bones, and mandibular bone shown in **(B)**. Scale bars represent 200 μm (left) and 500 μm (right). **(B,C)** High magnification representative images from coronal sections immunostained for Ankrd11 (red), with DAPI nuclear counterstain in blue from mandible and calvaria **(B)** as well as palatal shelves **(C)**. White dotted lines in **(B)** (mandible panels) indicate boundaries of mandibular bone indicated in **(A)**. Cranial/calvarial images are taken from comparable anatomic locations indicated in **(A)**. Images in **(B,C)** are presented from E14.5 and P0. Images in **(C)** are presented from E12.5, E13.5, E14.5, and P0. Graphic representation of Ankrd11 expression at E12.5 and E14.5 in right panels created in Adobe Illustrator. Blue represents Ankrd11 expression, with larger dots indicating relatively brighter regions. *n* = 3 from 3 litters. Scale bars in **(B)** represent 50 μm, except for E14.5 calvaria (200 μm). Scale bars in **(C)** represent 100 μm. ps, palatal shelf; t, tongue; tb, tooth bud; nc, nasal cavity; oc, oral cavity; es, epithelial seam; max, developing maxillary bone; m(m), molar (mesenchymal portion); m (e), molar (epithelial portion).

To gauge when and how *Ankrd11* could cause the above-described phenotypes, we performed expression analysis for *Ankrd11* at E13.5, E14.5, and P0 (Figure 4A). At E13.5, Ankrd11 expression was discrete in orofacial regions with clear expression in a mesenchymal condensation in the developing maxillary region (sphenoid wing). Some expression was noted in the developing palatal shelves and lining oral epithelium. Note the distinct expression in the developing forebrain at this stage. At E14.5, Ankrd11 expression was overall stronger, more widely expressed, and could be observed in several developing mesenchymal structures. Distinct expression was observed in ossification centers and at sites of bone development in the mandible and maxilla, as well as in pre-odontoblasts in the developing molars. Ankrd11 was also present in some cartilaginous structures (nasal septum, developing turbinates), parts of the tongue, and the midline epithelial seam of the fusing palate. In the eye, expression was prominent in the developing lens as well as in the anterior segment (Figure 4A). Extent and intensity of Ankrd11 expression waned by P0, with the majority of Ankrd11 at this point restricted to the oral epithelium. Expression in the mandibular and maxillary bones was strongly reduced or absent, as was expression in teeth. In the eye, expression was now restricted to the posterior segment (Figure 4A). This indicates dynamic, but discrete, expression of Ankrd11 throughout development of craniofacial structures.

This dynamic expression is nicely illustrated in the developing mandibular bone (Figure 4B), one of the earliest bones to mature in the craniofacial complex (Flaherty and Richtsmeier, 2018). While at E14.5, at the onset of ossification, Ankrd11 signal lined the forming mandibular bone, expression was negligible in the comparable region at P0 (Figure 4B). Similarly, in the cranial vault, Ankrd11 was localized to the forming ossification centers at E14.5, whereas at P0 weak expression was observed in cells lining the calvarial bone, but not in osteocytes within the mature bone (Figure 4B).

In comparison, Ankrd11 expression in the developing palatal shelves and the developing maxilla was more discrete. At E12.5, a mostly epithelial expression pattern was observed in palatal shelves (Figure 4C). A few Ankrd11-positive mesenchymal cells were evident in the buccal aspect toward its tip, as well as in its upper, dorsal aspects (Figure 4C). At E13.5 and E14.5, the epithelial and mesenchymal expression largely remained comparable to E12.5. Overall, at E14.5, Ankrd11 expression was strongest in epithelium of the newly forming nasal cavity, the oral epithelium, the developing maxillary bones, as well as in the fusing palatal shelf, restricted to the midline epithelial seam. With the exception of some epithelial structures, Ankrd11 expression at P0 was reduced in all of these places. Residual weak expression in presumptive bone-lining cells was noted (Figure 4C).

### *Ankrd11* Controls Proliferation in the Buccal Half of the Palatal Shelf

**FIGURE 5:**
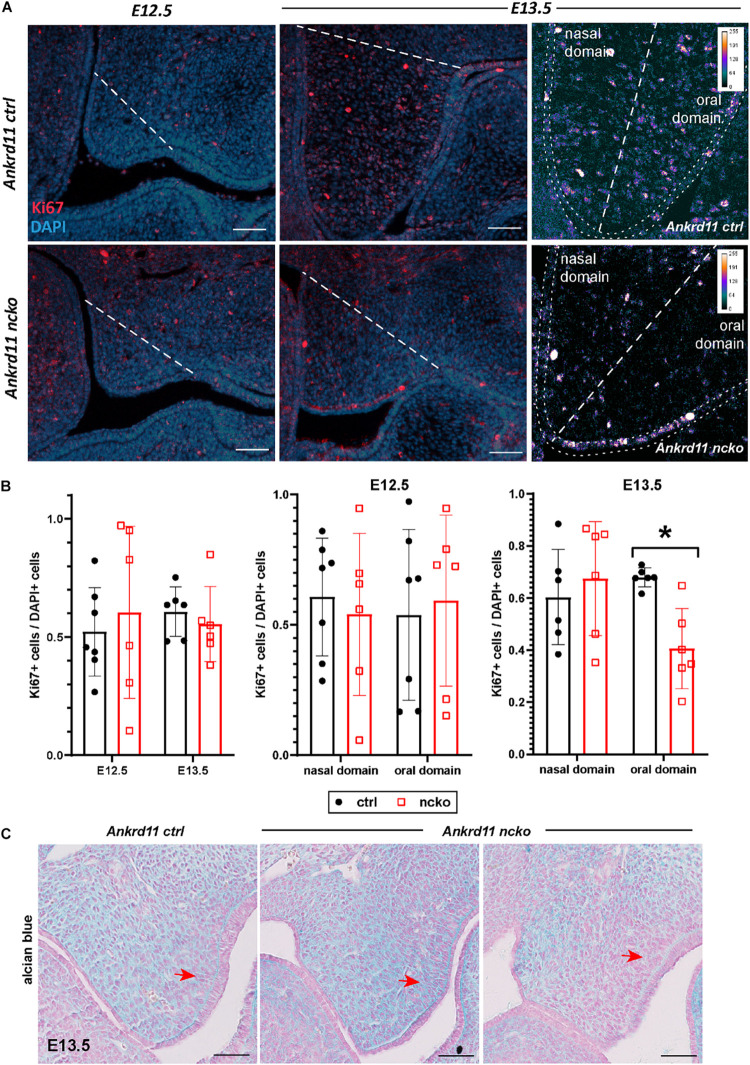
Palatal shelf proliferation is locally affected in Ankrd11^ncko^ embryos. **(A)** Representative images of palatal shelves at each age and timepoint immunostained for Ki67 (red). DAPI-labeled nuclei are in blue. White dotted line delineates region counted for quantification shown in **(B)**. White scale bars represent 50 μm. Right panels: Magnification of E13.5 palatal shelf showing heatmap of Ki67 expression using ImageJ. White dotted line separates the nasal and oral domains of the palatal shelf. Scale bars represent 50 μm. **(B)** Left panel: Quantification of Ki67-positive cells relative to DAPI-positive nuclei plotted for each genotype (Ankrd11^ctrl^ depicted in black and Ankrd11^ncko^ depicted in red at E12.5 and E13.5). Middle and right panel: Cell proliferation in nasal and oral domains respectively (independent two-sample *T*-test assuming unequal variances, minimum of six sections/genotype from minimum of five embryos per genotype, *p < 0.05, *n* = 6). **(C)** Alcian blue/nuclear fast red staining of E13.5 palatal shelves. Red arrow indicates mesenchymal cells lining epithelium on buccal side of shelf. *n* = 3/genotype, representative images shown. Scale bars represent 50 μm.

To assess the cause for the hypoplastic palatal shelves in Ankrd11^ncko^ embryos, we assessed apoptosis and proliferation. No significant apoptosis was observed at any of the time points between E12.5–E13.5 (Supplementary Figure 3). The overall ratio of Ki67-positive cells to DAPI-positive nuclei was comparable at E12.5 and E13.5 in Ankrd11^ncko^ palatal shelves when compared to Ankrd11^ctrl^ (Figures 5A,B). When shelves were separated into oral and nasal domains and a spatially restricted quantitative analysis was performed (Figure 5A, right panels), a 40% decrease in mesenchymal proliferation became obvious in the E13.5 oral domain (*n* ≥ 5 each, *p* < 0.05) (Figure 5B). To test for changes to cellular and extracellular organization, E13.5 palatal sections were stained with Alcian blue to identify distribution of glycosaminoglycans (GAGs). The overall organization and direction of cell orientation differs between Ankrd11^ncko^ and control shelves (Figure 5C). In addition, a disorganization of the mesenchymal cells lining the shelf epithelium was noticed (Figure 5C). These findings indicate that loss of *Ankrd11* has multiple subtle effects on palatal shelf organization and maturation, which likely underlie the cleft palate phenotype.

### *Ankrd11* Is Required for Normal Ossification of Intramembranous Bones

**FIGURE 6:**
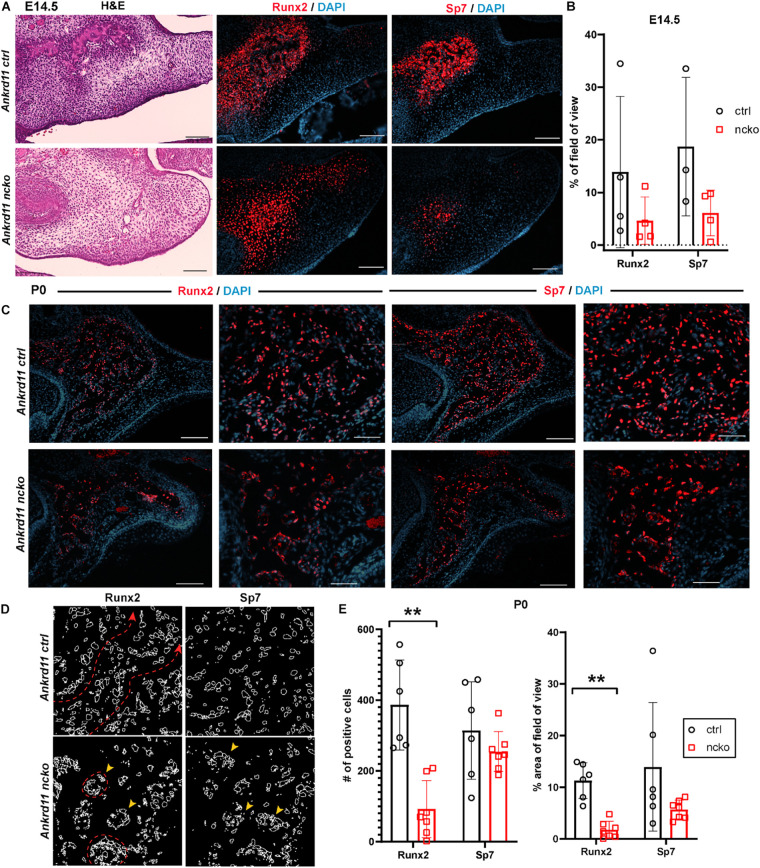
Bone formation in Ankrd11^ncko^ mice is dysregulated in late embryonic development. **(A)** Palatal region in E14.5 Ankrd11^ctrl^ (upper row) and Ankrd11^ncko^ (lower row) embryos H&E stained for orientation (left column) and immunostained for runt-related transcription factor 2 (Runx2) and Osterix (Sp7) (middle and right columns). Scale bars indicate 100 μm. **(B)** Quantification of % occupancy of field of view stained for Runx2 and Sp7 (independent two-sample *T*-test assuming unequal variances). **(C)** Low and high magnification images of palatal bone in P0 Ankrd11^ctrl^ (upper row) and Ankrd11^ncko^ (lower row) pups immunostained for Runx2 (left two columns) and Sp7 (right two columns). DAPI (blue) was used to counterstain nuclei. **(D)** Representation of individual cells expressing Runx2 and Sp7 generated using ImageJ showing altered cell distribution in the mutant. Red dotted line and arrowhead indicates extended trabeculae in control, whereas red circles and yellow arrowheads indicate more contained cellular clusters in the mutant. Scale bars in first and third columns represent 100 μm, and second and fourth represent 50 μm. **(E)** Quantification of Runx2 and Sp7 expression at P0 (left: number of positive cells; right: percent area per field of view) showing significant reduction of the area of Runx2 expression, whereas Sp7 expression domain was more contained but not statistically different at P0 (*n* = minimum of six sections form 3–5 pups/genotype, independent two-sample *T*-test assuming unequal variances, **p < 0.001).

To better understand the reduction in intramembranous ossification observed in Figure 2, protein expression of two master regulators of ossification, Runx2 and Sp7 (Hojo et al., 2016; Komori, 2017) was assessed within the field of view indicated in Figure 6A. At E14.5, expression of Runx2 and Sp7 quite homogenously outlined the growing maxillary bones in control embryos (Figure 6A, top row). In contrast, in Ankrd11^ncko^ mutant embryos, Runx2 and Sp7 expression was only strong in the center, becoming more diffuse toward the periphery. Sp7 expression was more contained in comparison to Runx2 (Figure 6A). Quantification of the respective expression domains indicated that expression of both Runx2 and Sp7 was more contained, however the differences in the area occupied did not reach statistical significance (Figure 6B).

**FIGURE 7:**
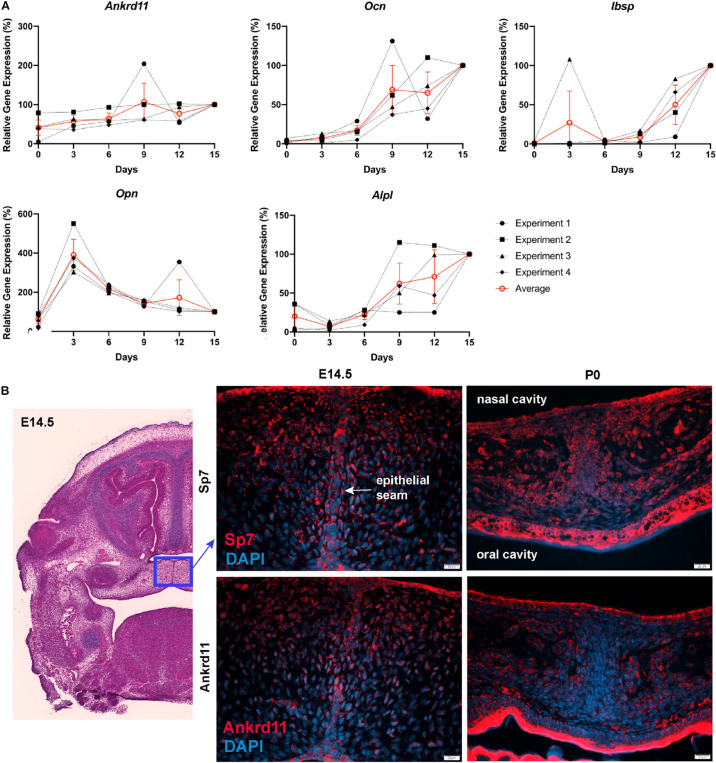
Ankrd11 is upregulated with bone induction. **(A)** RT-qPCR analysis of *Ankrd11*, *Ocn*, *Ibsp, Opn, and Alp1* mRNA expression during *in vitro* osteogenic differentiation of P6 calvarial osteoblast precursor cells from wild-type mice. Data was normalized to *36B4* housekeeping gene. Results are relative to d15 expression from four independent biological replicates with d15 values equated to 100%. **(B)** Low magnification of approximate location (blue box, H&E stain) of panels showing immunostaining for Osterix (Sp7, red, top row) and Ankrd11 (red, bottom row) in E14.5 and P0 Ankrd11^ctrl^ mice. Images indicate similar expression domains of Sp7 and Ankrd11 at embryonic stages with expression pattern varying at birth. DAPI (blue) was used to counterstain nuclei. *n* = 3. D, days; E, embryonic; P, postnatal.

Differences in expression became even more striking at P0, a time-point of significant maxillary bone growth and remodeling. Expression of Runx2 and Sp7 could be observed in osteoblasts lining the newly formed trabeculae in control neonates (Figure 6C, top row). In Ankrd11^ncko^, this trabecular pattern appeared disturbed. Expression of both Runx2 and Sp7 appeared patchy and locally more restricted to the small, sparse trabeculae in maxillary bone (Figure 6C, bottom row). Tracing the outlines of cells expressing Runx2 and Sp7 revealed the presence of extended, Runx2-positive cellular assemblies reminiscent of developing trabecula (Figure 6D, red dotted line), while the cellular structures in the mutant were more self-contained (Figure 6D, yellow arrowheads). Quantification indicated an 85% reduction of the Runx2-expression domain (*p* < 0.001) as well as 76% reduction in the number of Runx2-positive cells (*p* < 0.001). In contrast, despite a trend toward lower values, neither the number nor area of Sp7-positive cells were significantly different (Figure 6E). To test whether *Ankrd11* is indeed expressed and changes during osteoblast differentiation, we performed an *in vitro* time course of calvarial osteoblast differentiation. RT-qPCR analysis revealed that expression of *Ankrd11* changes dynamically over the course of osteogenic differentiation (Figure 7A). For reference, expression of several markers of osteoblast differentiation is shown: osteocalcin (*Ocn*), integrin binding sialoprotein *(Ibsp)*, Osteopontin (*Opn)*, alkaline phosphatase 1 (*Alp1*). This co-expression was mirrored *in vivo*, where Ankrd11, albeit more discrete, largely aligned with expression of Sp7 at E14.5, but not at P0 (Figure 7B).

### *Ankrd11* Is Required for Bone Maturation and Bone Remodeling

**FIGURE 8:**
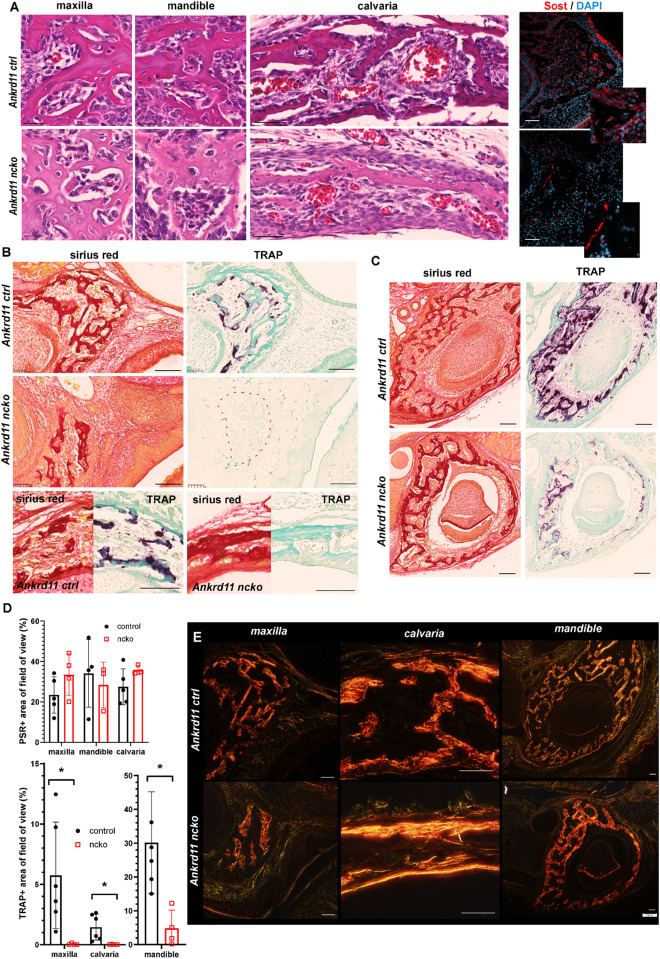
Bone maturation is delayed in Ankrd11^ncko^ mice. **(A)** Representative sections of maxillary, mandibular, and calvarial bone from Ankrd11^ctrl^ (top row) and Ankrd11^ncko^ (bottom row) mice at P0. Sections were stained simultaneously for H&E to reveal cell morphology and bone organization. Images were taken at anatomically matching locations. Images in right column depict sections immunostained for sclerostin (Sost, red) in the P0 maxillary bone, demonstrating a reduction in Sost^+^ cells in Ankrd11^ncko^ maxillary bone. DAPI (blue) was used to counterstain nuclei. Scale bars in first two columns represent 25 μm, and last two represent 50 μm. **(B)** Sirius red and tartrate-resistant acid phosphatase (TRAP) staining of maxilla and calvaria of Ankrd11^ctrl^ and Ankrd11^ncko^ mice at P0 on neighboring sections. Red dotted line outlines region of bone in Ankrd11^ncko^ maxilla with no TRAP-positive cells, indicating a reduction in remodeling. Calvarial images are taken at the same point midway between the eye and fontanelle. Scale bars represent 100 μm. **(C)** Sirius red and TRAP staining of the mandible in Ankrd11^ctrl^ and Ankrd11^ncko^ mice at P0 showing a decrease in TRAP-positive cells in the mandibular bone. Scale bars represent 100 μm. **(D)** Quantification of images shown in **(B,C)** (independent two-sample *T*-test assuming unequal variances, *p < 0.05, *n* = 5). **(E)** Polarized light images of sirius red stained maxilla, calvaria, and mandible in control and Ankrd11^ncko^ mice at P0 showing differences in extent of collagen fibril interconnection and orientation. Scale bars represent 50 μm.

The altered appearance of maxillary bones in Figure 7 prompted us to analyze maxilla, mandible, and calvaria trabeculae in more detail. In control mice, discrete trabeculae with widely interspaced osteocytes of mostly elongated appearance were readily identified (Figure 8A, top row). In contrast, trabeculae appeared less refined with a higher number of poorly aligned osteocytes evident in all bones from Ankrd11^ncko^ mice (Figure 8A, bottom row). Staining for the Wnt antagonist Sclerostin (Sost), a marker for mature osteocytes and an important regulator of bone remodeling (Winkler et al., 2003; Husain and Jeffries, 2017), revealed a strong reduction in Ankrd11^ncko^ mice (Figure 8A, right column), suggesting that osteocyte maturation is delayed and bone remodeling might be compromised. To better illustrate this, we stained coronal sections of the craniofacial complex with Picrosirius red to reveal collagen fiber networks and osteoclast-specific tartrate resistant alkaline phosphatase (TRAP). An overall reduction in trabeculation was seen in the maxilla, calvaria (Figure 8B), and mandible (Figure 8C). This was mirrored by a 99% reduction in TRAP-positive regions in the maxilla, 85% reduction in the mandible, and 99.5% reduction in the calvaria (Figure 8D bottom graph; *p* < 0.05) despite presence of comparable amounts of bone within the region of interest (Figure 8D top graph; n.s.). In the calvaria (Figure 8B, bottom panels), a single-layered bone was observed in Ankrd11^ncko^ mice indicative of reduced or defective bone remodeling. TRAP staining revealed an almost complete lack of bone resorption and, by extension, remodeling. Birefringent imaging under polarized light of picrosirius red-stained sections confirmed changes in trabeculation, interconnectivity, and crosslinking of collagen fibrils (Figure 8E). Thus, *Ankrd11* appears to be comparatively more important in bone growth and remodeling than the initial induction of intramembranous bone, despite its prominent expression in ossification centers.

## Discussion

Neural crest-specific deletion of *Ankrd11* reveals direct roles for *Ankrd11* in various aspects of craniofacial development. Our study focused on intramembranous bone formation of facial bones and the skull, as well as palate formation, as these structures are commonly affected in patients with KBG syndrome. Similarly to KBG syndrome, adult Ankrd11^nchet^ mice present with discrete phenotypic changes that match the descriptions of ‘triangular’ face, persistent anterior fontanelle, and palatal abnormalities in humans. These observations complement the phenotypic description of heterozygous “Yoda” mice, in which an ENU-induced point-mutation in the C-terminus of *Ankrd11* leads to a shorter and wider face, decreased bone mineral density, incomplete closure of the interfrontal suture, and persistent opening of the anterior fontanelle (Barbaric et al., 2008). While both heterozygous Yoda and Ankrd11^nchet^ mutants are valuable to relate effects of *Ankrd11* mutations on development, they do not allow for attribution of systemic consequences and overall developmental delay to discrete roles of *Ankrd11*. In contrast, relating phenotypic consequences of homozygous, cell-specific deletion of *Ankrd11* to cellular expression of *Ankrd11* elucidates the involvement of *Ankrd11* in a precise and applicable manner. In this study, we focused on neural crest and craniofacial development. Ankrd11^ncko^ neonates exhibit a triangular shape face, cleft palate, midfacial hypoplasia, retrognathia, bone defects including an expanded anterior fontanelle, hypoplastic frontal bones, and a delay in bone maturation. Ankrd11^ncko^ die at birth, which underscores the overall importance of *Ankrd11* in neural crest-derived structures. Our findings on intramembranous ossification and palate development indicate that *Ankrd11* controls neural crest-derived cell progenitor proliferation or differentiation in a spatio-temporal manner, without affecting apoptosis. These data support and extend previous reports, where knockdown of *Ankrd11* in neural precursors decreases their proliferation and resulting number of differentiated neurons during murine cortical development (Gallagher et al., 2015). Our findings that *Ankrd11* impinges on intramembranous bone formation and maturation allow comparison to 3D morphometric analysis in both mice and humans to make precise conclusions on functional impact. Thus, the homozygous deletion of *Ankrd11* in a tissue-specific manner provides a powerful approach to model the KBG syndrome phenotypes in mice. Furthermore, this approach highlights how discrete perturbations in chromatin and epigenetic control can compromise tissue development and function long-term.

### *Ankrd11* Is Expressed Discretely in a Variety of Craniofacial Tissues

Neural crest-specific deletion reveals the broad involvement of *Ankrd11* during craniofacial development. In line with this, Ankrd11 shows restricted and dynamic expression in many craniofacial tissues. At E13.5 and E14.5, Ankrd11 is localized to discrete parts of the eye, bones, teeth, cartilage, and several muscular components. At a later developmental stage, expression in these regions is comparatively low and is primarily restricted to oral epithelium. Some expression in muscular compartments and olfactory bulb was noted in line with Gallagher et al. (2015). Relating expression to phenotypic manifestations allows strong predictions on the involvement of *Ankrd11* in the development of these different structures. For instance, Ankrd11 expression is noted from E13.5 onward in mesenchymal condensations and at E14.5 in ossification centers. This implies that the bony defects observed in Ankrd11^ncko^ mice might precipitate from differences during bone induction, although some of the defects such as osteocyte maturation and remodeling manifest only at much later stages. A similar pattern is seen in the maxillary and calvarial bones, where Ankrd11 is strongly expressed early in development, yet the impact on bone maturation and remodeling is noted only in much later stages when Ankrd11 expression is markedly reduced. It was previously suggested that development of bone involves epigenetic mechanisms, which might in part be controlled by lineage-specific transcription factors such as Sp7/Osterix (Park-Min, 2017). Whereas induction of ossification centers appears not to be compromised, significant differences are observed with respect to growth and maturation of these ossification centers. Expression domains of Runx2 and Sp7 are differentially affected. Thus, our study illustrates how expression of these two master genes are variably affected by loss of *Ankrd11*. At present, it is unclear if the weak expression of Ankrd11 seen in more mature bone indicates a continuing requirement for *Ankrd11* to maintain established epigenetic signatures, or whether it indicates ongoing, not-yet-understood *de novo* genomic fine-tuning.

### *Ankrd11* Is Required for Expansion of Intramembranous Ossification Centers

Both 3D morphological as well as phenotypic analysis indicate that *Ankrd11* is particularly important for intramembranous ossification. Establishment of ossification centers appears not to be compromised in the Ankrd11^ncko^ embryos; however, they universally fail to fully expand. This was not due to changes in proliferation or apoptosis (Supplementary Figure 3) but rather appeared to be the consequence of other, not fully understood mechanisms affecting specification, maturation, cell orientation, or migration of the induced bone-forming cells. In this light, Yoda mice or mice with *Ankrd11* knockdown in cortical progenitors display aberrant positioning of neurons in the developing cortex linked to dysregulated epigenetic mechanisms (Gallagher et al., 2015). Together with our craniofacial data, this could underscore that control of cell migration may be a common mechanism.

Intramembranous calvarial bone development generally occurs in two stages: (1) development of bone primordia from mesenchymal condensations, e.g. anterior-superior of eye for the frontal bone (beginning at E12.5), and (2) expansion, e.g. of the frontal bone front to cover the top of the skull (Yoshida et al., 2008). Defects in either or both of these stages result in insufficient bone formation. Our results suggest that loss of *Ankrd11* predominantly affects extension of the bone via growth at the osteogenic front and bone remodeling. The frontal bone insufficiency seen in Ankrd11^ncko^ mice mirrors the phenotype observed in the Beetlejuice mouse, which carries a mutation in *Prickle1* (Prickle1^bj/bj^) (Wan et al., 2018). Prickle1^bj/bj^ mice also have hypoplastic frontal bones, an expanded anterior fontanelle, and frequently cleft palate. The Prickle1 mutation is thought to affect a *Wnt*/Planar cell polarity (*Wnt*/PCP) signaling pathway. Apoptosis and proliferation are not affected, but unlike the *Ankrd11* mutant, a notable decrease in Sp7 alongside minimal changes to Runx2 expression is observed (Wan et al., 2018). Mutations in core components of the *Wnt*/PCP pathway cause Robinow syndrome which, like KBG syndrome, results in a wider midface and shorter stature. Notwithstanding these phenotypic similarities, changes in Ankrd11^ncko^ mice are likely caused by inappropriate epigenetic regulation in differentiating osteoblasts. Histones associated with several gene loci for key osteogenic factors including Runx2, Sp7, and Alp undergo dynamic acetylation changes throughout the ossification process (Zhang et al., 2015). Of interest, Runx2 interacts directly with HDAC3, a histone deacetylase previously shown to directly interact with Ankrd11 (Schroeder et al., 2004; Zhang et al., 2004; Gallagher et al., 2015). It is thus possible that *Ankrd11* and *Runx2* participate in common gene regulatory networks to set up osteoblast differentiation during craniofacial bone development. Potential changes in epigenetic signatures were not investigated as part of this study, because craniofacial structures are too heterogeneous following different developmental kinetics, posing significant challenges for the analysis of subtle epigenetic changes.

### Delayed Maturation of Bone in Ankrd11^ncko^ Mice

Bone remodeling begins soon after bone formation. In Ankrd11^ncko^ mice, bone remodeling was severely compromised. The only notable remodeling occurred in the mandible, but even there it was dramatically reduced. Osteocytes appeared to retain an immature phenotype, evidenced by their increased numbers, plump morphology, apparent failure to align along stress/force lines, and lack of Sclerostin (Sost) expression, a characteristic of immature osteocytes. This could be the consequence of a delay in bone formation or an intrinsic defect in osteocyte differentiation. As Ankrd11 is not expressed in osteocytes (Figure 4B), this phenotype is likely caused by abnormal osteoblast differentiation, possibly involving inappropriate epigenetic osteoblast programming or specification as discussed earlier.

Immature osteocytes lack the exquisite mechanosensory properties of mature osteocytes. The primary cilium has been suggested to play an important role in bone mechanotransduction (Temiyasathit and Jacobs, 2010). Inability to sense or respond to mechanical cues would manifest as failure to align along stress lines, increased cell number, and failure to induce bone remodeling (exemplified by loss of TRAP staining). On the other hand, osteocyte maturation is dependent on mineralization, as the transition to mature osteocytes is triggered by differences in mechanosensing of mineralized and unmineralized matrix (Irie et al., 2008). Changes to ordered mineralization in Ankrd11^ncko^ bones could equally explain the failure in osteocyte maturation and associated bone remodeling. Osteoclasts are related to monocytes and are of hematopoietic origin. Neural crest-specific *Ankrd11* deletion would not directly affect these cells. The defect in remodeling is therefore most likely due to defective mechanosensing or defective communication with osteoclasts, underlining interconnection of these systems.

While lack of Sost expression could reflect a failure of osteocyte maturation, it must be noted that *Sost* itself is under epigenetic control. HDAC5 deficiency results in increased Sost expression, impaired osteogenesis, and low bone density (Wein et al., 2015). Sost activity-blocking antibodies are an approved treatment modality for osteoporosis aiming to reduce bone resorption. A more detailed analysis will be required to carefully dissect the cause and consequence of these bone-related phenotypes.

### A Role for *Ankrd11* in Palate Development

In contrast to bone primordia, the pattern of Ankrd11 expression in the palate is relatively discrete. It also differs from the often broad expression domains described for many transcription factors and signaling molecules in the developing palatal shelves (He et al., 2011; Danescu et al., 2015). This restricted Ankrd11 expression was seen at all stages from E12.5-E14.5 and could be attributed to discrete cells organizing palate development, or a tight window of expression governing reprogramming. Thus, Ankrd11 might be involved in the spatial organization of the palatal shelf, possibly regulating the expression of a growth factor or its receptors, facilitating growth of the palatal shelf secondarily. Indeed, changes in cellular distribution were observed.

Furthermore, an asymmetric pattern of proliferation was observed at E13.5, with a decrease in the buccal half of the shelf. The affected region roughly corresponds to the gene expression domain of the Hedgehog signaling receptor Patched (Ptch) (Xiong et al., 2009), raising the possibility that Ankrd11 somehow intersects with Hh signaling and cilia. Hedgehog and Wnt signaling are indeed both critical for cilia maturation, and functional cilia are required for normal palatal development (Brugmann et al., 2010; Nakaniwa et al., 2019). Recently, *Ankrd11* was directly associated with ciliopathies (Breslow et al., 2018). The interplay between Hedgehog and Wnt signaling and chromatin remodeling by Ankrd11 may provide important clues for understanding both palate and bone phenotypes and will be investigated in future studies.

### Clinical Implications

Mesh analysis of four patients with KBG syndrome revealed hypoplastic mid- and lower face and enlarged upper face. This is largely mimicked in Ankrd11^nchet^ and Ankrd11^ncko^ mice (Figures 1, 2). Given the stunted intramembranous ossification in the Ankrd11^ncko^ mouse, we propose that the expansion of the upper 1/3 of the face is a consequence of underdeveloped midfacial bones as well as intramembranously formed extensions of the cranial base – the pterygoid wings. The resultant narrower cranial base and midface would necessitate increased cranial bone growth to accommodate the expanding brain, leading to a triangular face. The calvaria would not be able to meet the increased demand in bone growth (as they are compromised in bone formation themselves) resulting in a persistent anterior fontanelle.

Patients with KBG syndrome show various accompanying craniofacial anomalies. Macrodontia, dental crowding, and hypo/oligodontia are often observed, necessitating invasive jaw surgery (Ockeloen et al., 2015; Low et al., 2016; Morel Swols and Tekin, 2018). Our results and expression analysis suggest that these malformations may be due to abnormal tooth and jaw development. Moreover, speech and feeding difficulties are frequently observed in KBG syndrome patients (Ockeloen et al., 2015; Low et al., 2016; Morel Swols and Tekin, 2018). The anomalies in palate development, more severe in Ankrd11^ncko^ mice but evident also in Ankrd11^nchet^ mice, can provide a structural explanation for these observations. High-arched palate or submucosal cleft, considered milder variants of defective palate development, lead to similar problems. Thus, our results will precipitate further investigations into the underpinnings of the various phenotypic craniofacial anomalies associated with *ANKRD11*.

*ANKRD11* is one of the most disrupted genes in monogenic neurodevelopmental disorders with *de novo* mutations (Wilfert et al., 2017; Satterstrom et al., 2020). Yet, most patients with KBG syndrome are not diagnosed until 20–30 years of age, if at all (personal communication with Drs. Charlotte Ockeloen, Tjitske Kleefstra, and Peter Kannu). The gold standard for diagnosis of KBG syndrome is genetic testing to detect *ANKRD11* variants or 16q24.3 deletion involving the gene. Specific craniofacial features such as persistent anterior fontanelle, submucosal or high-arched palate, macrodontia, and reduced bone mineral density are currently only suggestive of KBG syndrome and clinical phenotypic criteria for proper diagnosis remain poorly defined (Morel Swols and Tekin, 2018). This study confirms the power of 3D morphometric analysis to assist with syndrome diagnosis (Hallgrímsson et al., 2020) and illustrates the power of disease modeling in the mouse to unearth the underlying cellular basis for the malformation. Indeed, many craniofacial features observed in KBG syndrome (Ockeloen et al., 2015; Low et al., 2016; Morel Swols and Tekin, 2018) are recapitulated in the Ankrd11^ncko^ mouse. This study has begun to unravel some of the unknowns of *Ankrd11*-mediated regulation of craniofacial development and has allowed assessment of craniofacial malformations as a result of *Ankrd11* deletion or loss-of-function. Systematic assessment of cataloged craniofacial features in patients with KBG syndrome can potentially drive further understanding of clinical penetrance and future establishment of definitive phenotypic diagnostic criteria to achieve earlier differential diagnosis, as well as clarify the impact of *ANKRD11* variants of unknown significance (VUS). Similarly, studies in other organ systems often involved in KBG syndrome will further clarify the role of *Ankrd11* in epigenetic and genomic control of tissue and organ development.

### Summary

Our phenotypic characterization of conditional ablation of *Ankrd11* in the murine neural crest revealed novel roles of *Ankrd11* in craniofacial development, specifically in intramembranous bone formation and palate development. Our results will help improve clinical assessment of patients with KBG syndrome based on craniofacial phenotypic diagnosis, particularly early in life. Ultimately, early diagnosis and better understanding of *ANKRD11* function will assist with genetic counseling for patients with KBG syndrome and their affected families.

## Data Availability Statement

The raw data supporting the conclusions of this article will be made available by the authors, without undue reservation.

## Ethics Statement

The studies involving human participants were reviewed and approved by Institutional Review Boards at the University of Calgary, University of Colorado, Denver, and the University of California, San Francisco. Written informed consent to participate in this study was provided by the participants’ legal guardian/next of kin. The animal study was reviewed and approved by Research Ethics Office at the University of Alberta (Animal Care and Use Committee) under AUP1149 and AUP2527.

## Author Contributions

DR performed the experiments, analyzed the results, and wrote the manuscript. PB performed the experiments, analyzed the results, and edited the manuscript. HL and MV-G performed the experiments and analyzed the results. S-TD and DGo performed the preliminary experiments. AW and TF performed the supporting experiments and mouse line management. NS and SE generated the Ankrd11^fl/fl^ mice. MV-G, JA, and BH performed the morphometric analyses of human subjects and mice and edited the manuscript. DGr and AV conceived the study, analyzed the results, wrote and edited the manuscript, and provided funding. All the authors contributed to the article and approved the submitted version.

## Conflict of Interest

The authors declare that the research was conducted in the absence of any commercial or financial relationships that could be construed as a potential conflict of interest.

## Supplementary Material

Supplementary Figure 1 Landmarks used for mesh morphometric analysis. Sixty-eight anatomical landmarks placed on micro-CT volumes of the cranium using the MINC toolkit.

Supplementary Figure 2 Ankrd11^ncko^ mice are born with a macroscopically recognizable craniofacial phenotype. Left column: Ankrd11^ctrl^ P0 mice. Right column: Ankrd11^ncko^ P0 mice. **(A,B)** Lateral view of head, with a white arrow indicating layer of skin covering eye, incomplete in Ankrd11^ncko^ mice. Ankrd11^ncko^ mice also have a growth deficiency in the midface and retrognathia. **(C,D)** Superior view of head. Ankrd11^ncko^ appears paler, with a more domed head shape. **(E,F)** View of the snout from the front. White arrow indicates black pigment that is absent in Ankrd11^ncko^ mice. **(G,H)** View of dissected palate, viewed from below. Left side is anterior. Asterisk highlights cleft palate in Ankrd11^ncko^ mouse. **(I,J)** View of dissected mandible and tongue, viewed from above. Left side is anterior. Note tongue (central, spanning left to right in image) is thinner and shorter in Ankrd11^ncko^ mouse. Consistent amongst three mice from different litters.

Supplementary Figure 3 Cleaved Caspase 3 (CC3) dependent apoptosis does not differ between Ankrd11^ctrl^ and Ankrd11^ncko^ palatal shelves. Representative immunofluorescent staining for cleaved caspase 3 (CC3) on coronal paraffin sections (green). Nuclei are counterstained with DAPI (red). *n* = 3 from 3 l. Scale bar represents 50 μm on all images.

Supplementary Table 1 Antibodies used for immunofluorescent staining.

Supplementary Table 2 Oligonucleotide primer pairs used for quantitative RT-PCR.

## Abbreviations

*Ankrd11*: Ankyrin Repeat Domain 11
*ncko*: neural crest knockout
*nchet*: neural crest heterozygous deletion
E__: embryonic day __
*36B4*: acidic ribosomal phosphoprotein P0
*Alpl*: alkaline phosphatase
CBP: CREB-associated factor
*CC3*: cleaved caspase 3
CREB: cAMP-response element-binding protein
EDTA: ethylenediaminetetraacetic acid
ENU: *N*-ethyl -*N*-nitrosourea mutagenesis
Flp: flippase
HBSS: Hanks’ Balanced Salt Solution
HDAC3/5: histone deacetylase 3/5
*Hh*: Hedgehog
*Ibsp*: integrin-binding sialoprotein
Ki67: Marker of Proliferation Ki-67
*Ocn*: osteocalcin
*Opn*: osteopontin
P/CAF: p300/CBP-associated acetyltransferase complex
P0: postnatal day 0
PFA: paraformaldehyde
*Prickle1*: Prickle Planar Cell Polarity Protein 1
RT-qPCR: reverse transcriptase quantitative polymerase chain reaction
*Runx2*: Runt-related transcription factor 2
*Sost*: sclerostin
*Sp7*: osterix
TRAP: tartrate-resistant acid phosphatase
*Wnt1*: Wnt family member 1
*Wnt*/PCP: Wnt planar cell polarity
αMEM: minimum essential medium α
μCT: micro-computed tomography.
