# Supplementary figures and images for: The Chromatin Regulator Ankrd11 Controls Palate and Cranial Bone Development
Source: Front Cell Dev Biol. 2021 Apr 29;9:645386. doi: 10.3389/fcell.2021.645386 (PMC8117352; doi:10.3389/fcell.2021.645386)

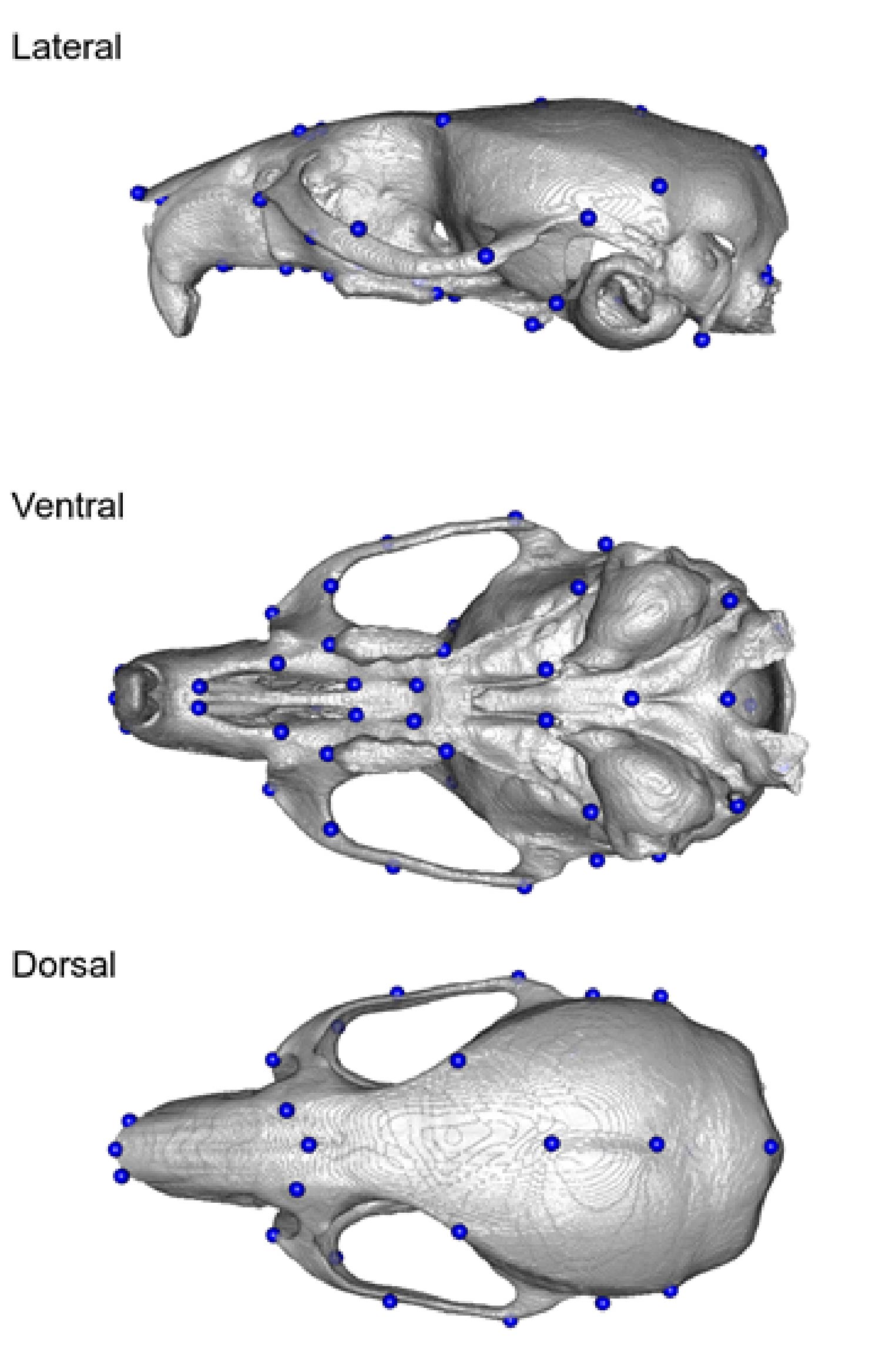

Supplement: Supplementary Figure 1 — Landmarks used for mesh morphometric analysis. Sixty-eight anatomical landmarks placed on micro-CT volumes of the cranium using the MINC toolkit. [file Image_1.JPEG]

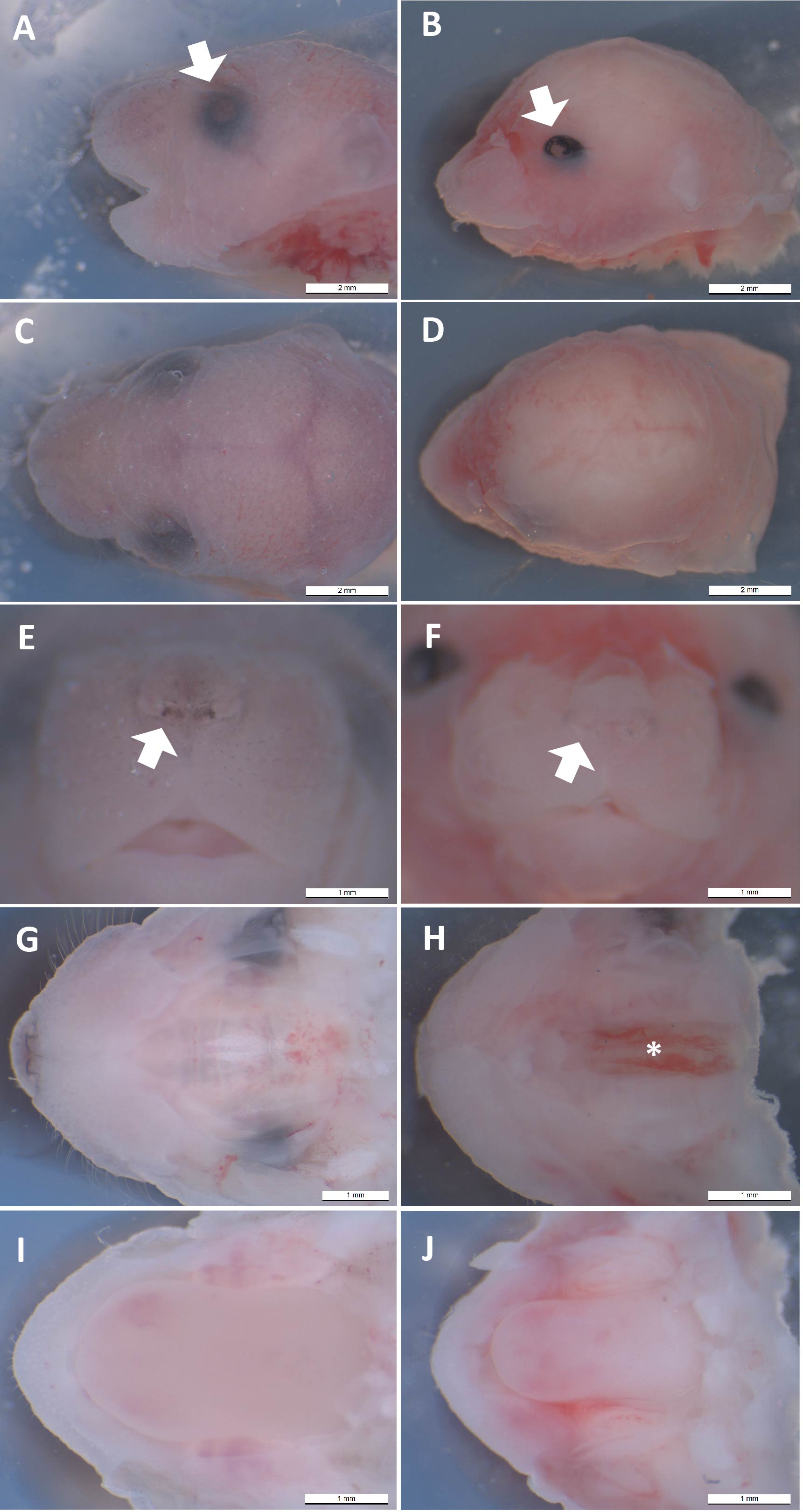

Supplement: Supplementary Figure 2 — Ankrd11ncko mice are born with a macroscopically recognizable craniofacial phenotype. Left column: Ankrd11ctrl P0 mice. Right column: Ankrd11ncko P0 mice. (A,B) Lateral view of head, with a white arrow indicating layer of skin covering eye, incomplete in Ankrd11ncko mice. Ankrd11ncko mice also have a growth deficiency in the midface and retrognathia. (C,D) Superior view of head. Ankrd11ncko appears paler, with a more domed head shape. (E,F) View of the snout from the front. White arrow indicates black pigment that is absent in Ankrd11ncko mice. (G,H) View of dissected palate, viewed from below. Left side is anterior. Asterisk highlights cleft palate in Ankrd11ncko mouse. (I,J) View of dissected mandible and tongue, viewed from above. Left side is anterior. Note tongue (central, spanning left to right in image) is thinner and shorter in Ankrd11ncko mouse. Consistent amongst three mice from different litters. [file Image_2.JPEG]

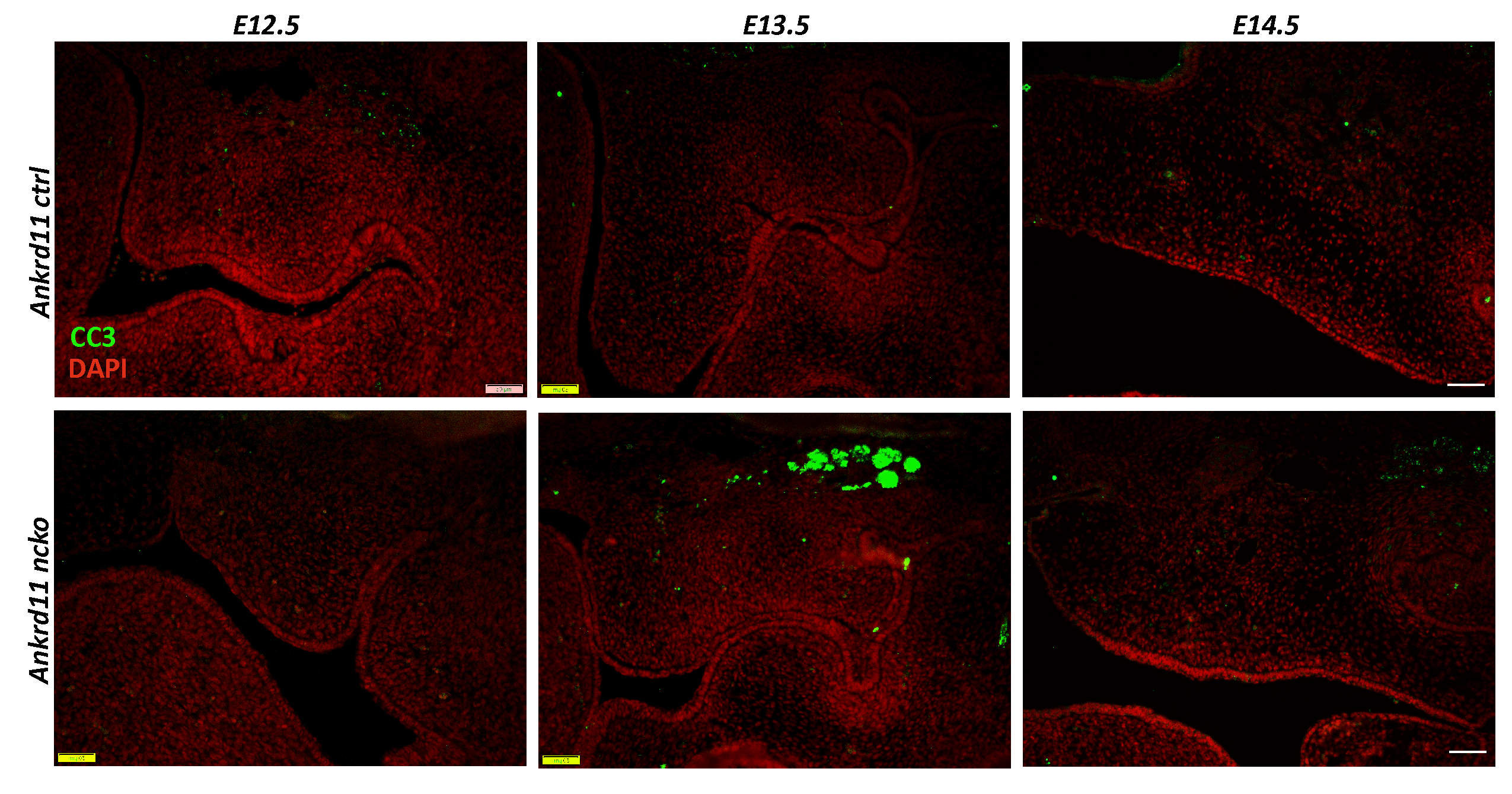

Supplement: Supplementary Figure 3 — Cleaved Caspase 3 (CC3) dependent apoptosis does not differ between Ankrd11ctrl and Ankrd11ncko palatal shelves. Representative immunofluorescent staining for cleaved caspase 3 (CC3) on coronal paraffin sections (green). Nuclei are counterstained with DAPI (red). n = 3 from 3 l. Scale bar represents 50 μm on all images. [file Image_3.tif]
