# Supplementary material for: The Chromatin Regulator Ankrd11 Controls Palate and Cranial Bone Development
Source: Front Cell Dev Biol. 2021 Apr 29;9:645386. doi: 10.3389/fcell.2021.645386 (PMC8117352; doi:10.3389/fcell.2021.645386)
Supplement: Supplementary Table 1 — Antibodies used for immunofluorescent staining. [file Data_Sheet_1.PDF]

Antibody name	***Catalogue #***	***Primary antibody concentration***	***Secondary antibody***	***Secondary antibody concentration***
Ankyrin repeat domain 11 peptide 2 (Ankrd11)	Lab-made†	1:400	donkey anti-rabbit polyclonal 647	1:1000
Marker of Proliferation Ki-67 (Ki67)	Ebioscience B-5698	1:300	goat anti-mouse IgG_1 647	1:1000
Cleaved Caspase 3 (CC3)	Cell Signaling 9664s	1:100	donkey anti-rabbit polyclonal 647	1:1000
Runt-related transcription factor 2 (Runx2)	Santa Cruz SC-2903	1:100	goat anti-mouse IgG_1 647	1:1000
Osterix (Sp7)	Abcam ab94744	1:800	donkey anti-rabbit polyclonal 647	1:1000

† α-ANKRD11 polyclonal antibody raised against a synthetic peptide representing residues 326-341 of the ANKRD11 sequence, conjugated to KLH and affinity-purified on Sepharose beads coupled with the same peptide. Antibody was a generous gift from Dr. D. Callen (Neilsen et al., 2008).

Gene	***Abbreviation***	***Primer pair***
Acidic ribosomal phosphoprotein P0	36B4	GTG TGT CTG CAG ATC GGG TA<br>CAG ATG GAT CAG CCA GGA AG
Ankyrin repeat domain 11	Ankrd11	TAAAGGAGTGTGGGTTCGAGA<br>TCAAGGGTGGACTTGTCACTT
Osteocalcin	Ocn	ATAGCTCGTCACAAGCAGGG<br>TGACAAAGCCTTCATGTCCA
Integrin-binding sialoprotein	Ibsp	CAGGGAGGCAGTGACTCTTC<br>AGTGTGGAAAGTGTGGCGTT
Osteopontin	Opn	AATCTCCTTGCGCCACAGAA<br>GCAGTGACGGTCTCATCAGA
Alkaline phosphatase	Alpl	GGCAGCGTCAGATGTTAATTG<br>ACTGCGCTCCTTAGGGCT
